# Racial and Gender Disparities in Incidence of Lung and Bronchus Cancer in the United States: A Longitudinal Analysis

**DOI:** 10.1371/journal.pone.0162949

**Published:** 2016-09-29

**Authors:** Mohammad A. Tabatabai, Jean-Jacques Kengwoung-Keumo, Gabriela R. Oates, Juliette T. Guemmegne, Akinola Akinlawon, Green Ekadi, Mona N. Fouad, Karan P. Singh

**Affiliations:** 1 School of Graduate Studies and Research, Meharry Medical College, Nashville, TN 37208, United States of America; 2 Department of Mathematical Sciences, Cameron University, Lawton, OK 73505, United States of America; 3 Division of Preventive Medicine, University of Alabama at Birmingham, Birmingham, AL 35294, United States of America; 4 Department of Economics, University of New Mexico, MSC 05 3060, Albuquerque, NM 87131, United States of America; 5 Department of Computing and Technology, Cameron University, Lawton, OK 73505, United States of America; 6 Comprehensive Cancer Center, University of Alabama at Birmingham, Birmingham, AL 35294, United States of America; National Health Research Institutes, TAIWAN

## Abstract

**Background:**

Certain population groups in the United States carry a disproportionate burden of cancer. This work models and analyzes the dynamics of lung and bronchus cancer age-adjusted incidence rates by race (White and Black), gender (male and female), and prevalence of daily smoking in 38 U.S. states, the District of Columbia, and across eight U.S. geographic regions from 1999 to 2012.

**Methods:**

Data, obtained from the U.S. Cancer Statistics Section of the Centers for Disease Control and Prevention, reflect approximately 77% of the U.S. population and constitute a representative sample for making inferences about incidence rates in lung and bronchus cancer (henceforth lung cancer). A longitudinal linear mixed-effects model was used to study lung cancer incidence rates and to estimate incidence rate as a function of time, race, gender, and prevalence of daily smoking.

**Results:**

Between 1999 and 2012, age-adjusted incidence rates in lung cancer have decreased in all states and regions. However, racial and gender disparities remain. Whites continue to have lower age-adjusted incidence rates for this cancer than Blacks in all states and in five of the eight U.S. geographic regions. Disparities in incidence rates between Black and White men are significantly larger than those between Black and White women, with Black men having the highest incidence rate of all subgroups. Assuming that lung cancer incidence rates remain within reasonable range, the model predicts that the gender gap in the incidence rate for Whites would disappear by mid-2018, and for Blacks by 2026. However, the racial gap in lung cancer incidence rates among Black and White males will remain. Among all geographic regions, the Mid-South has the highest overall lung cancer incidence rate and the highest incidence rate for Whites, while the Midwest has the highest incidence rate for Blacks. Between 1999 and 2012, there was a downward trend in the prevalence of daily smokers in both genders. However, males have significantly higher rates of cigarette smoking than females at all time points. The highest and lowest prevalence of daily smoking are found in the Mid-South and New England, respectively. There was a significant correlation between lung cancer incidence rates and smoking prevalence in all geographic regions, indicating a strong influence of cigarette smoking on regional lung cancer incidence rates.

**Conclusion:**

Although age-adjusted incidence rates in lung cancer have decreased throughout the U.S., racial and gender disparities remain. This longitudinal model can help health professionals and policy makers make predictions of age-adjusted incidence rates for lung cancer in the U.S. in the next five to ten years.

## Introduction

Lung and bronchus cancer is the second most commonly diagnosed cancer (excluding non-melanoma skin cancer) in the United States [1]. Approximately 7.7% of men and 6.3% of women will be diagnosed with this cancer during their lifetime. The risk factors for lung and bronchus cancer (henceforth lung cancer) are tobacco use, family history, and environmental and occupational exposures, such as second-hand smoke, radon, and asbestos [2]. According to the Centers for Disease Control and Prevention, cigarette smoking is the number one risk factor for lung cancer, linked to about 80% to 90% of lung cancers in the United States [3]. Racial/ethnic and socio-demographic differences exist in the prevalence of cigarette smoking, with higher use among males, especially Blacks males, persons with lower education, and those with annual household income less than $20,000 [4]. Similarly, lung cancer incidence rates vary substantially by gender, race/ethnicity, socioeconomic status, and geography, in large part because of differences in cigarette-smoking patterns [5]. Lung cancer incidence is higher among Black males, people of lower socioeconomic status, and persons living in the South [6].

Although reduced tobacco use and increased prevention and early detection efforts have improved lung cancer outcomes for both men and women, disparities remain [7, 8]. The National Cancer Institute defines cancer disparities as adverse differences in cancer incidence, prevalence, mortality, survivorship, and burden among specific population groups [9]. Several studies have reported pronounced racial/ethnic disparities in lung cancer in the United States [10–13]._ Others have demonstrated that adjusting for socioeconomic status virtually eliminates racial/ethnic differences in stage-adjusted lung cancer mortality [14]. In this study, we investigate disparities in incidence rates of lung cancer by race (Black/White), gender, and prevalence of daily smoking in 38 U.S. states, the District of Columbia, and the eight U.S. geographic regions between 1999 and 2012.

Because of variation in confounders of lung cancer incidence at state, regional, and group levels, the longitudinal analysis was applied as a linear mixed-effects model. Mixed-effects models are powerful tools in longitudinal studies because they allow for a simultaneous estimation of fixed and random coefficients. Random coefficients capture variation among and within elements, whereas fixed parameters improve the predictions through calibration of various dynamics of the model. When the longitudinal response is discrete, generalized linear [15] and nonlinear [16] mixed-effects models are more appropriate for relating changes in the mean response to covariates [17]. Tabatabai et al. [18, 19] were first to use a longitudinal hyperbolastic mixed-effects type II model in cervical cancer research. They analyzed disparities in cervical cancer mortality rates between White and Black women in 13 U.S. states between 1975 and 2010 and attributed racial disparities to differences in socioeconomic factors, such as education and poverty levels, as well as to screening and treatment modalities. In this paper, we use a longitudinal linear mixed-effects model to analyze disparities in lung cancer incidence in the United States and establish a predictive model that estimates lung cancer incidence rate as a function of time, race, gender, and prevalence of daily smoking.

## Methods

Lung cancer incidence data for 1999–2012 were obtained from the U.S. Department of Health and Human Services Cancer Statistics Section, available through the Centers for Disease Control and Prevention WONDER database [20]. The data set included 38 U.S. states (Alabama, Arizona, Arkansas, California, Colorado, Connecticut, Delaware, Florida, Georgia, Illinois, Indiana, Iowa, Kansas, Kentucky, Louisiana, Maryland, Massachusetts, Michigan, Minnesota, Mississippi, Missouri, Nebraska, Nevada, New Jersey, New York, North Carolina, Ohio, Oklahoma, Oregon, Pennsylvania, Rhode Island, South Carolina, Tennessee, Texas, Virginia, Washington, West Virginia, and Wisconsin) and the District of Columbia. The total number of observations in this study is 2,169. The choice of states was based on data availability for both racial groups. We doubled the sample size by separating Blacks from Whites. The combined data reflect approximately 77% of the U.S. population and constitute a representative sample for making inferences about lung cancer incidence rates in the U.S.

Smoking prevalence rates were obtained from Dwyer-Lindgren et al. [21], who performed a comprehensive study of smoking prevalence in U.S. counties using data of over 4 million adults from the Behavioral Risk Factor Surveillance System (BRFSS) for 1996–2012 [22]. The authors utilized the BRFSS data and applied validated small-area estimation techniques to estimate daily cigarette smoking prevalence for U.S. counties. Using the county data for male and female smokers from 1999 to 2012, we estimated the percent daily smokers for all 38 states and District of Columbia.

We used a mixed-effects model because of observed variability in lung cancer incidence rates at state and regional levels. After initial screening of the data, including tests for linearity, we selected the following linear mixed-effects model:

$$Y_{ij}=\beta_{0j}+{Β}_{j}\prime X_{ij}+\varepsilon_{ij},$$ (1)

where the response variable *Y*_*ij*_ is the *i*^*th*^ lung cancer incidence rate in the *j*^*th*^ state at time *T*_*ij*_ (*i* = 1,2,…,*n*_*j*_), and *n*_*j*_ is the number of observations from the *j*^*th*^ state. *β*_0*j*_, *β*_4*j*_ are unknown state-specific regression coefficients, and *β*_0*j*_ = *β*_0_ + *U*_0*j*_ and *β*_4*j*_ = *β*_4_ + *U*_1*j*_ are used to explain the observed variability between the states with respect to daily smoking. *β*_0_, *β*_4_ are unknown regression parameters, and *U*_0*j*_ and *U*_1*j*_ are state-specific random effects, where *U*_0*j*_ expresses how much the intercept of state *j* which is denoted by *β*_0*j*_ deviates from the global intercept *β*_0_, and *U*_1*j*_ expresses how much the slope of the percent of daily smokers for state *j* denoted by *β*_4*j*_ deviates from the global slope *β*_4_. The vector of explanatory variables *X*_*ij*_ is defined as

$$X_{ij}=\left[\begin{array}{c} \begin{array}{c} \begin{array}{c} R_{ij}\\ G_{ij}\\ T_{ij} \end{array}\\ S_{ij} \end{array}\\ G_{ij}T_{ij}\\ G_{ij}T_{ij}R_{ij}\\ G_{ij}S_{ij}\\ S_{ij}G_{ij}R_{ij}\\ S_{ij}G_{ij}R_{ij}T_{ij} \end{array}\right]$$

where the variables *R*_*ij*_, *G*_*ij*_, *T*_*ij*_, and *S*_*ij*_ represent race, gender, time, and percentage of daily smokers together with their associated interactions.

For each observation *i* in state *j*, *T*_*ij*_ represents time point in year, with *T*_*ij*_ = 1 corresponding to the year 1999 and *T*_*ij*_ = 14 corresponding to the year 2012, and *R*_*ij*_ and *G*_*ij*_ being indicator variables representing race and gender, respectively. Specifically, *R*_*ij*_ = 1 is used for White race and *G*_*ij*_ = 1 for male individual. Likewise, *R*_*ij*_ = 0 if the incidence rate *Y*_*ij*_ is measured for Black individual and *G*_*ij*_ = 0 if the incidence rate *Y*_*ij*_ is measured for a female. ${Β}_{j}=\left[\begin{array}{c} \begin{array}{c} \begin{array}{c} \begin{array}{c} \begin{array}{c} \beta_{1}\\ \beta_{2}\\ \beta_{3} \end{array}\\ \beta_{4j} \end{array}\\ \beta_{5} \end{array}\\ \beta_{6} \end{array}\\ \beta_{7}\\ \beta_{8}\\ \beta_{9} \end{array}\right]$ (*B*′_*j*_ being the transpose of *B*_*j*_), *β*_4*j*_ represents the slope for percent of daily smokers and is the random slope associated with *S*_*ij*_. The random vector $\left[\begin{array}{c} U_{0j}\\ U_{1j} \end{array}\right]$ is the vector of random effects, and *β*_0_, *β*_1_,…,*β*_9_ are assumed to be fixed parameters. The random effects vector $\left[\begin{array}{c} U_{0j}\\ U_{1j} \end{array}\right]$ has a bivariate normal distribution with mean vector $\left[\begin{array}{c} 0\\ 0 \end{array}\right]$ and variance-covariance matrix $V=\left[\begin{array}{c} \sigma_{11}\;\sigma_{12}\\ \sigma_{21}\;\sigma_{22} \end{array}\right]$, where *σ*_11_ represents variance in intercepts between states, *σ*_22_ represents variance in slopes of the variable race between states, and *σ*_12_ = *σ*_21_ is the covariance between random intercepts and random slopes. The error term *ε*_*ij*_ is normally distributed with mean 0 and constant variance *σ*^2^.

Adding interaction terms to the model expands the understanding of the association between lung cancer incidence rates and model variables and the interpretations of the variable coefficients. Eq 1 can be presented in an expanded form as

$$Y_{ij}=\beta_{0j}+\beta_{1}R_{ij}+\beta_{2}G_{ij}+\beta_{3}T_{ij}+\beta_{4j}S_{ij}+\beta_{5}G_{ij}T_{ij}+\beta_{6}G_{ij}T_{ij}R_{ij}+\beta_{7}G_{ij}S_{ij}+\beta_{8}G_{ij}S_{ij}R_{ij}+\beta_{9}G_{ij}S_{ij}R_{ij}T_{ij}+\varepsilon_{ij}$$ (2)

By replacing *R*_*ij*_ by 0 and *G*_*ij*_ by 1 in Eq 2, we obtain the following equation for the lung cancer incidence rate for Black males: *Y*_*ij*_ = (*β*_0*j*_ + *β*_2_) + (*β*_3_ + *β*_5_)*T*_*ij*_ + (*β*_4*j*_ + *β*_7_)*S*_*ij*_ + *ε*_*ij*_

A one-percent increase in the Black male daily cigarette smokers would result in an increase of (*β*_4*j*_ + *β*_7_) cases per 100,000 in the incidence of lung cancer for Black males. For White males, replacing *R*_*ij*_ by 1 and *G*_*ij*_ by 1 in Eq 2 gives the following equation of lung cancer incidence rate: *Y*_*ij*_ = (*β*_0*j*_ + *β*_1_ + *β*_2_) + (*β*_3_ + *β*_5_ + *β*_6_)*T*_*ij*_ + (*β*_4*j*_ + *β*_7_ + *β*_8_ + *β*_9_*T*_*ij*_)*S*_*ij*_ + *ε*_*ij*_

A one-percent increase in the White male daily cigarette smokers would result in an increase of (*β*_4*j*_ + *β*_7_ + *β*_8_ + *β*_9_*T*_*ij*_) cases per 100,000 in the incidence of lung cancer for White males.

Similarly, by replacing the corresponding numbers for race and gender using Eq 2, we obtain the equations for White females and Black females.

According to the Surveillance, Epidemiology, and End Results (SEER) data, the formula for incidence rate is:

AIR=100,000*Sumof(eachagespecificrate*eachstandardpopulationweight),

where

$$age\;specific\;rate=\frac{Number\;of\;incidents\;in\;age\;group}{Population\;of\;age\;group},$$

and

$$standard\;population\;weight=\frac{Population\;for\;age\;group}{Sum\;of\;populations\;for\;all\;age\;groups}.$$

Age-adjusted incidence rates were calculated with age distribution ratios from the year 2000 standard million population and are shown per 100,000 people. For brevity, in the remainder of this paper we use incidence rates to mean age-adjusted incidence rates. Computer-based analyses were performed with SAS version 9.3, SPSS version 23, and Mathematica version 10.

## Results

Using the model described above and SAS PROC NLMIXED features, we obtained the model parameter values. **Table 1**shows the estimates of coefficients and the associated standard errors. An estimate of the unstructured variance covariance matrix for the longitudinal linear mixed-effects model is given by:

$$\hat{V}=\left[\begin{array}{cc} 66.8774 & -3.8328\\ -3.8328 & 0.3518 \end{array}\right]$$

**Table 1:** Summary of model parameter estimates, standard errors, t-values, p-values, 95% confidence interval, and gradient.

Parameter	Estimate	Standard Error	t-Value	P-Value	LowerCI	UpperCI	Gradient
*β*_0_	24.9292	2.7493	9.07	< .0001	19.3587	30.4998	-1.06107
*β*_1_	1.9664	.2190	8.98	< .0001	1.5227	2.4101	.010065
*β*_2_	4.2692	1.7734	2.41	< .0212	.6760	7.8625	.15547
*β*_3_	.2389	.04537	5.26	< .0001	.1469	.3308	.044569
*β*_4_	1.6056	.1498	10.72	< .0001	1.3020	1.9092	.131981
*β*_5_	-1.1680	.05452	-21.42	< .0001	-1.2784	-1.0575	.014155
*β*_6_	-1.7093	.1548	-11.04	< .0001	-2.0229	-1.3956	-.00931
*β*_7_	2.3255	.07530	30.88	< .0001	2.1730	2.4781	.057392
*β*_8_	-1.0845	.02271	-47.75	< .0001	-1.1305	-1.0385	-.00557
*β*_9_	.1087	.00843	12.90	< .0001	.09165	.1258	-.13998
*σ*_11_	66.8774	5.8111	11.51	< .0001	55.1030	78.6518	-1.19139
*σ*_22_	.3518	.04313	8.16	< .0001	.2644	.4392	-.11709
*σ*_12_	-3.8328	.3914	-9.79	< .0001	-4.6259	-3.0396	.10909
*σ*^2^	130.60	1.2598	103.67	< .0001	128.05	133.16	.010062

*Note*: Level of significance, ∝ = 0.05; degrees of freedom, *df* = 37; *CI* = *Confidence Interval*

As seen in **Table 1,** race, gender, time, percentage of daily smokers, and the interaction variables are all significant predictors of lung cancer incidence rate. Based on the parameter estimates from Table 1, the estimated predictive equation for lung cancer is:

Y=24.9292+1.9664R+4.2692G+0.2389T+1.6056S−1.1680GT−1.7093GTR+2.3255GS−1.0845RGS+0.1087RGST (3)

The estimated incidence rate for Black males is:

Y=29.1921−0.9291T+3.9311S

and the corresponding equation for White males is:

Y=31.15852−2.6384T+2.8466S+0.1087ST

The equation for White males reveals interaction between time *T* and percent daily smokers *S*. This indicates that the rate of change of lung cancer incidence with respect to change in daily smoking is different at different time points. For Black males, this rate is constant at the level 3.9311; for White males, the rate is a linear function of time *T* and is estimated as (2.8466+0.1087*T*). The results indicate that among Black males, a 1% increase in the percent of daily smokers will increase the Black male lung cancer incidence rate by approximately 3.9311 cases per 100,000. For White and Black females, this rate remains constant. For females, we have approximated the percent daily smokers as a function of time by

S=18.848−0.424T (4)

For males, the estimated equation is

S=23.156−0.448T (5)

Replacing *S* in Eq 3 using Eq 4 results in the following equation for the incidence rate of females as a function of race and time:

Y=55.1853+1.9664Race−0.441874Time (6)

Similarly, replacing *S* in Eq 3 using Eq 5 results in the following equation for the incidence rate of males as a function of race and time:

Y=120.221−2.69023Time+Race[−23.1463+1.29361Time−0.0486976(Time)^2] (7)

**Fig 1:**
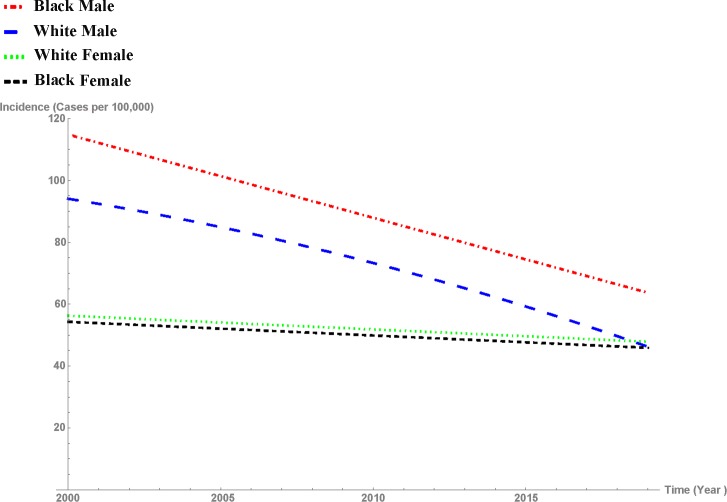
Predictive curves of lung cancer incidence rates by race and gender (cases per 100,000).

Using Eqs 6 and 7 for Blacks and Whites would result in four equations for the incidence rates of lung cancer as a function of time for Black and White males and females. **Fig 1**depicts these four graphs.

This predictive model accurately assesses the fluctuating trends of lung cancer incidence rates from 1999 to 2012. Although there is no universally acceptable coefficient of determination (R^2^) for longitudinal mixed-effects models, we use the Xu [23] formula defined as

$$R^{2}=1-\frac{Variance\;for\;full\;model}{Variance\;for\;null\;model}.$$

For our model, R^2^ is 0.82. Assuming that lung cancer incidence rates remain within reasonable range, the model predicts that by mid-2018, the gender gap in the incidence rate for Whites would disappear. At that time, the common incidence rate for Whites regardless of gender would be approximately 48 cases per 100,000. By year 2026, the gender gap in the incidence rate for Blacks would disappear as well. However, the racial gap in lung cancer incidence among males will not disappear in the near future. For year 2013, the model estimates of lung cancer for Black males, White males, White females, and Black females are 89.87, 65.17, 50.52, and 48.56, respectively. These estimates are consistent with the estimates of the Centers for Disease Control and Prevention [24].

**Fig 2:**
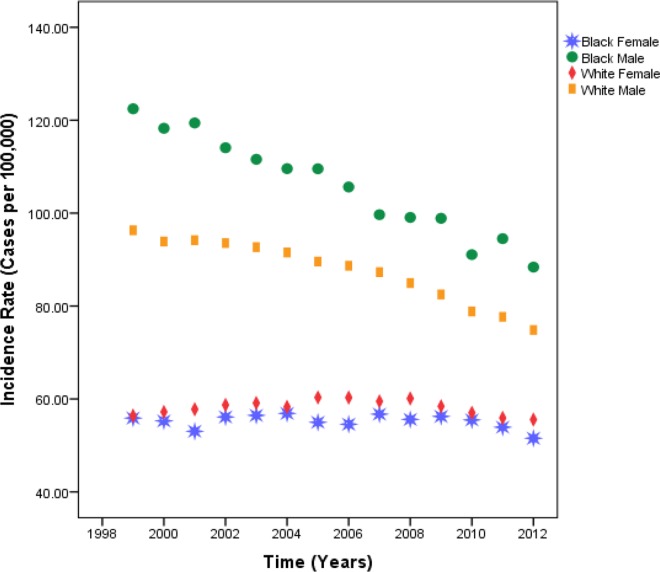
Estimates of lung cancer mean incidence rates by race and gender across all U.S. geographic regions, 1999–2012.

Table 2 gives the mean incidence rates for Blacks and Whites from 1999 to 2012. **Fig 2**shows that from 1999 to 2012, Whites continued to have lower burden of lung cancer incidence than Blacks. The mean incidence rates in lung cancer for Blacks and Whites decreased from 90.72 to 70.46 and from 76.34 to 65.20, respectively.

**Table 2:** Lung cancer incidence rates by race, 1999–2012 (cases per 100,000).

Race	Year	Mean	Std Dev	CV	Minimum	Lower Quartile	Median	Upper Quartile	Maximum
**Black**	1999	90.72	38.44	42.37	34.10	57.30	86.80	124.00	183.10
	2000	87.73	36.63	41.75	34.80	55.20	84.45	114.20	173.30
	2001	88.23	38.10	43.18	33.40	53.80	85.50	122.70	178.50
	2002	85.97	34.94	40.63	38.80	56.50	74.15	118.50	163.40
	2003	85.15	33.45	39.28	33.10	55.30	79.80	112.00	163.50
	2004	83.97	32.28	38.45	36.70	54.55	76.85	114.40	155.80
	2005	83.38	34.13	40.93	38.80	54.70	73.10	109.70	189.80
	2006	81.15	31.67	39.03	35.10	53.50	72.90	107.40	149.30
	2007	77.93	29.30	37.60	38.10	52.70	72.30	99.70	180.20
	2008	77.62	28.90	37.23	34.00	53.70	73.20	100.40	156.20
	2009	77.57	28.09	36.21	34.70	53.50	71.30	100.10	159.80
	2010	73.28	24.38	33.27	34.90	50.55	72.25	93.55	140.10
	2011	73.95	26.18	35.41	33.40	50.20	70.20	97.70	128.10
	2012	70.46	23.49	33.34	34.60	48.10	66.15	90.60	122.50
**White**	1999	76.34	23.74	31.09	40.40	57.00	70.90	95.00	142.40
	2000	75.55	22.46	29.72	43.80	56.80	70.85	92.90	139.60
	2001	75.98	22.37	29.44	39.90	57.90	72.20	91.85	146.10
	2002	76.14	21.04	27.63	46.10	59.20	70.60	90.80	139.90
	2003	75.90	20.56	27.09	42.90	59.10	69.55	93.10	135.10
	2004	74.92	21.53	28.74	29.60	59.20	66.95	89.50	139.00
	2005	74.95	19.85	26.49	34.70	60.20	68.60	88.60	129.20
	2006	74.49	19.25	25.84	41.90	60.20	68.10	85.60	131.70
	2007	73.40	18.78	25.59	30.00	60.80	66.05	85.70	126.40
	2008	72.52	18.68	25.76	26.90	60.90	69.25	83.70	127.10
	2009	70.47	17.61	24.98	24.40	58.90	66.05	80.70	123.90
	2010	67.93	16.41	24.16	42.00	56.50	63.35	79.20	125.40
	2011	66.79	16.77	25.12	24.10	56.40	62.35	78.85	113.60
	2012	65.20	15.12	23.19	37.90	54.85	63.10	74.50	111.90

To further explore differences in lung incidence rates between the two racial groups, we computed mean incidence rates by gender (**Table 3**and **Table 4**). Within each racial group, we observed pronounced gender disparities in lung cancer incidence. Black men have incidence rates about 1.8 times higher than those for Black women, while White men have incidence rates approximately 1.4 times higher than those for White women.

**Table 3:** Lung cancer incidence rates for Blacks by gender, 1999–2012.

Gender	Year	Mean	Std Dev	CV(%)	Minimum	Lower Quartile	Median	Upper Quartile	Maximum
**Female**	1999	55.89	14.31	25.60	34.10	47.40	57.00	63.30	86.80
	2000	55.28	12.84	23.22	34.80	48.05	54.55	61.10	87.90
	2001	53.04	12.31	23.20	33.40	42.10	53.00	61.00	81.10
	2002	56.12	11.73	20.90	38.80	45.70	55.90	66.65	76.50
	2003	56.47	13.41	23.75	33.10	44.90	55.00	67.30	86.20
	2004	56.89	14.38	25.27	36.70	46.20	54.50	66.20	95.90
	2005	54.99	11.05	20.09	38.80	44.90	54.50	63.90	74.40
	2006	54.56	12.39	22.71	35.10	45.10	53.20	68.50	82.00
	2007	56.74	12.57	22.16	38.10	48.30	53.10	65.30	90.40
	2008	55.60	16.01	28.79	34.00	45.40	54.10	64.30	117.10
	2009	56.25	12.76	22.68	34.70	47.50	53.50	63.50	88.60
	2010	55.49	13.86	24.98	34.90	45.70	51.10	64.80	95.40
	2011	53.93	12.72	23.58	33.40	45.80	50.25	61.60	82.30
	2012	51.54	10.50	20.37	34.60	44.50	47.70	58.65	76.60
**Male**	1999	122.46	22.39	18.28	64.50	108.40	121.35	138.90	183.10
	2000	118.26	22.50	19.03	78.90	100.60	114.15	133.50	173.30
	2001	119.41	22.62	18.94	82.60	100.00	120.90	129.20	178.50
	2002	114.08	24.42	21.41	61.50	95.00	115.75	128.50	163.40
	2003	111.58	22.72	20.36	67.40	97.10	111.65	124.60	163.50
	2004	109.58	21.76	19.86	59.70	91.10	114.40	124.10	155.80
	2005	109.54	26.25	23.97	67.00	92.10	109.30	124.10	189.80
	2006	105.64	22.93	21.71	58.10	87.00	106.50	118.40	149.30
	2007	99.67	25.35	25.44	49.60	79.20	99.90	115.30	180.20
	2008	99.07	21.53	21.74	52.90	82.20	99.70	110.30	156.20
	2009	98.89	22.43	22.68	57.50	86.20	100.10	110.80	159.80
	2010	91.07	19.03	20.90	49.10	74.70	92.30	105.90	140.10
	2011	94.51	19.63	20.77	60.10	73.70	97.70	105.70	128.10
	2012	88.39	17.48	19.78	56.30	72.00	89.75	98.50	122.50

**Table 4:** Lung cancer incidence rates for Whites by gender, 1999–2012.

Gender	Year	Mean	Std Dev	CV(%)	Minimum	Lower Quartile	Median	Upper Quartile	Maximum
**Female**	1999	56.38	7.84	13.91	40.40	50.80	57.00	62.20	72.10
	2000	57.21	7.28	12.73	43.80	52.10	57.10	61.30	76.30
	2001	57.78	7.76	13.42	39.90	52.95	57.90	61.75	75.40
	2002	58.72	6.83	11.63	46.10	53.30	59.20	62.80	76.10
	2003	59.12	7.24	12.25	42.90	53.60	59.10	62.50	77.00
	2004	58.31	8.13	13.94	29.60	53.30	59.50	63.10	76.80
	2005	60.32	8.66	14.35	34.70	56.70	61.10	63.10	80.70
	2006	60.31	8.18	13.57	41.90	56.00	60.50	65.00	80.40
	2007	59.49	8.24	13.86	30.00	55.30	60.90	63.00	80.50
	2008	60.11	9.31	15.49	26.90	56.50	61.00	64.50	82.60
	2009	58.43	8.86	15.17	24.40	56.30	59.40	63.00	80.70
	2010	57.01	7.37	12.92	42.20	53.90	57.10	60.90	82.90
	2011	55.90	8.95	16.00	24.10	53.50	57.50	59.90	81.30
	2012	55.57	7.49	13.47	37.90	52.00	56.45	58.60	78.70
**Male**	1999	96.30	16.18	16.80	66.40	87.20	95.00	107.50	142.40
	2000	93.89	16.67	17.75	53.70	84.80	92.90	104.30	139.60
	2001	94.18	16.52	17.54	68.70	82.80	91.85	104.25	146.10
	2002	93.56	15.05	16.09	69.00	84.00	90.80	104.10	139.90
	2003	92.69	15.02	16.20	62.10	83.50	93.10	104.80	135.10
	2004	91.54	17.51	19.12	44.20	81.40	89.50	102.70	139.00
	2005	89.58	16.87	18.83	41.70	81.10	88.60	101.30	129.20
	2006	88.67	16.46	18.57	56.50	80.00	85.60	100.50	131.70
	2007	87.30	15.82	18.12	58.50	77.10	85.70	99.20	126.40
	2008	84.94	17.43	20.52	31.50	75.50	83.70	95.80	127.10
	2009	82.50	15.88	19.25	46.30	70.80	80.60	94.60	123.90
	2010	78.84	15.72	19.93	42.00	70.10	77.70	89.50	125.40
	2011	77.68	15.70	20.22	35.70	68.10	76.90	88.30	113.60
	2012	74.84	14.72	19.67	40.30	66.90	73.25	84.60	111.90

Gender disparities in lung cancer incidence are thus more severe in the Black population than in the White population. In the 14-year time period, the lung cancer incidence rates for females of either race have been lower than the incidence rate for their male counterparts and remain stable throughout the study period. Over time, males of either gender have decreasing incidence rate, but Black males have higher incidence curve compared to White males.

Tables 2–4 show the estimated standard deviations, coefficient of variation (CV), and five-number summary. The CV, which is the ratio of the standard deviation to the mean, was computed to control for the differences in the mean mortality rates for each race in the 38 U.S. states and the District of Columbia. The CV for a single variable aims to describe the dispersion of the variable in a way that does not depend on the variable’s measurement unit; the higher the CV is, the greater the dispersion of the variable. Among the 38 states and the District of Columbia, Kentucky had the highest incidence rate for all four subgroups: Black males, Black females, White males, and White females. Delaware had the lowest incidence rate among White females, Florida had the lowest incidence rate among Black females, and Nevada had the lowest incidence rate for Black males.

There is evidence that lung cancer incidence rates vary by geographic region [25, 26]. We grouped the 39 elements in our investigation (38 states and the District of Columbia) into eight U.S. geographic regions to assess regional differences in lung cancer incidence rates by race. The geographic regions were New England (Connecticut, Massachusetts, and Rhode Island), Mid-Atlantic (District of Columbia, New Jersey, New York, and Pennsylvania), Midwest (Illinois, Indiana, Iowa, Kansas, Michigan, Minnesota, Missouri, Nebraska, Ohio, and Wisconsin), Pacific Coast (California, Oregon, and Washington), Rocky Mountain (Colorado and Nevada), Mid-South (Alabama, Arkansas, Kentucky, Louisiana, Mississippi, Tennessee), South (Delaware, Florida, Georgia, Maryland, North Carolina, South Carolina, Virginia, and West Virginia), and Southwest (Arizona, Oklahoma, and Texas). **Table 5**shows the lung cancer incidence rates for Blacks and Whites by U.S. geographic region. Among Blacks, the highest mean incidence rate (92.92) was observed in the Midwest, and the lowest mean incidence rate (63.19) in New England. The Mid-South had the highest coefficient of variation (42.89%) while the Rocky Mountain had the lowest coefficient of variation (30.89%). Among Whites, the highest mean incidence rate (86.24) was observed in the Mid-South, and the lowest mean incidence rate (63.20) in the Mid-Atlantic. The Mid-South had the highest coefficient of variation (29.65%), while New England had the lowest coefficient of variation (17.72%).

**Table 5:** Summary statistics of lung cancer incidence rates for Blacks and Whites in 8 U.S. Geographic Regions, 1999–2012 (cases per 100,000).

U.S. Region	Race	Mean	Std Dev	CV(%)	Minimum	Lower Quartile	Median	Upper Quartile	Maximum
**Mid-Atlantic**	Black	75.35	26.25	34.84	39.30	52.50	72.50	96.20	145.00
	White	63.20	17.12	27.09	24.10	54.20	59.70	80.00	91.70
**Mid- South**	Black	87.45	37.51	42.89	33.10	50.05	92.35	119.45	173.30
	White	86.24	25.57	29.65	48.70	60.75	83.75	107.65	146.10
**Midwest**	Black	92.92	31.83	34.26	35.10	65.60	85.35	117.50	189.80
	White	70.90	17.49	24.66	44.60	56.55	65.00	87.65	108.90
**New England**	Black	63.19	19.59	31.01	33.40	45.80	62.35	79.20	101.50
	White	73.87	13.09	17.72	53.70	62.90	69.35	84.65	105.10
**Pacific Coast**	Black	82.48	30.07	36.46	45.50	58.30	74.15	103.10	183.10
	White	64.34	11.69	18.18	41.50	56.75	62.60	72.15	92.10
**Rocky Mountain**	Black	63.29	19.55	30.89	38.00	48.15	59.35	72.70	114.20
	White	64.14	16.17	25.21	41.60	47.50	66.70	75.70	102.10
**South**	Black	74.43	30.06	40.39	34.30	45.90	71.30	100.00	154.40
	White	77.67	19.36	24.93	51.50	59.95	72.45	94.70	129.20
**Southwest**	Black	78.43	31.34	39.97	35.20	53.35	62.45	112.45	143.70
	White	68.69	19.16	27.89	43.80	52.65	64.55	81.40	115.00

In five U.S. geographic regions (Mid-Atlantic, Mid-South, Midwest, Pacific Coast, and Southwest), the mean incidence rates for Blacks were higher than those for Whites during the period 1999–2012. In the remaining three regions (New England, Rocky Mountain, and South), this trend was reversed over time. During 1999–2012, the coefficients of variation for Whites in all regions were smaller than those for Blacks, indicating that lung cancer incidence rates for Whites were more homogeneous across U.S. regions.

We also analyzed gender disparities in lung cancer incidence rates within each racial group by geographic region. During the 14-year period, incidence rates for men were higher than those for women in both racial groups. There was an insignificant racial difference in the incidence rates of women but pronounced racial disparities in the incidence rates of men. Overall, during the 14-year period, Black men continued to have higher incidence rates in all U.S. regions.

**Table 6**shows that the Mid-South region had the highest mean incidence rate (86.85) of all regions, while the Rocky Mountain had the lowest mean incidence rate (63.72), regardless of race and gender. The Mid-South also had the highest coefficient of variation, while New England had the lowest coefficient of variation. These findings prompted us to investigate further the dynamics of incidence rates in the Mid-South.

**Table 6:** Summary statistics of lung cancer incidence rates for Whites in 8 U.S. geographic regions, 1999–2012 (cases per 100,000).

U.S. Region	Mean	Std Dev	CV (%)	Minimum	Lower Quartile	Median	Upper Quartile	Maximum
**Mid-Atlantic**	69.28	22.94	33.11	24.10	53.85	65.30	84.45	145.00
**Mid-South**	86.85	32.05	36.90	33.10	57.95	88.30	112.00	173.30
**Midwest**	81.63	27.76	34.01	35.10	60.40	72.40	98.10	189.80
**New England**	69.25	17.01	24.57	33.40	58.45	68.05	82.25	105.10
**Pacific Coast**	72.59	23.75	32.72	41.50	57.30	64.60	81.90	183.10
**Rocky Mountain**	63.72	17.86	28.03	38.00	47.90	60.15	74.70	114.20
**South**	76.07	25.24	33.19	34.30	56.10	71.90	97.30	154.40
**Southwest**	73.56	26.35	35.83	35.20	53.00	63.85	92.30	143.70

We applied our longitudinal linear mixed-effects models to the Mid-South region, comprised of six states: Alabama, Arkansas, Kentucky, Louisiana, Mississippi, and Tennessee. **Table 7**shows that in 1999 the difference between mean incidence rates for Blacks and Whites was about 3/100,000. The highest mean incidence rate was recorded in Kentucky, while the lowest in Alabama (**Table 8**). The racial gap fluctuated but eventually narrowed down to almost zero in 2012, suggesting that the Mid-South region is successful in eradicating racial disparities in lung cancer incidence rates. However, gender disparities persisted throughout the 14-year period. Of note, the mean incidence rate for White women was higher than that for Black women, while the opposite was observed for men (**Table 9**).

**Table 7:** Lung cancer incidence rates for Blacks and Whites in the Mid-South, 1999–2012 (cases per 100,000).

Race	Year	Mean	Std Dev	CV(%)	Minimum	Lower Quartile	Median	Upper Quartile	Maximum
**Black**	1999	93.10	44.88	48.20	34.10	50.70	99.80	124.00	150.20
	2000	95.65	52.97	55.38	34.80	46.50	91.65	136.00	173.30
	2001	88.99	43.17	48.52	33.40	45.50	99.00	125.40	138.70
	2002	90.48	47.47	52.46	40.40	44.90	87.15	127.95	163.40
	2003	85.91	40.83	47.53	33.10	48.20	92.85	123.75	139.90
	2004	93.48	41.10	43.97	42.20	51.55	105.75	126.90	155.80
	2005	91.37	40.41	44.23	39.90	50.70	91.65	124.45	153.30
	2006	87.56	37.32	42.62	39.50	49.85	93.80	117.20	145.00
	2007	85.87	35.10	40.87	43.90	51.05	89.35	117.95	140.20
	2008	88.61	38.50	43.45	38.00	54.10	92.65	117.55	156.20
	2009	87.32	37.02	42.40	38.80	51.90	99.40	115.55	143.80
	2010	83.64	34.78	41.59	36.50	50.65	89.35	110.85	140.10
	2011	82.12	34.08	41.51	42.20	47.20	87.90	112.65	127.90
	2012	78.73	29.95	38.04	34.60	48.85	83.95	107.05	112.00
**White**	1999	90.98	37.11	40.78	48.70	58.00	90.35	116.10	142.40
	2000	91.85	34.76	37.84	53.50	59.50	92.00	114.50	139.60
	2001	92.31	33.50	36.29	54.10	62.00	91.15	116.00	146.10
	2002	89.31	30.92	34.62	57.10	59.05	89.10	110.60	139.90
	2003	87.43	28.35	32.43	56.20	59.20	90.50	108.45	135.10
	2004	89.16	28.71	32.21	56.60	60.90	91.60	110.70	139.00
	2005	87.10	25.46	29.23	58.80	61.65	90.55	107.75	129.20
	2006	86.89	26.98	31.05	56.00	60.55	90.30	108.80	131.70
	2007	87.10	25.24	28.98	58.60	62.70	87.85	108.15	126.40
	2008	87.90	24.47	27.84	60.60	63.10	90.35	106.95	127.10
	2009	84.17	23.27	27.64	59.40	60.05	84.90	101.90	123.90
	2010	82.38	22.87	27.76	56.80	59.15	88.15	98.65	125.40
	2011	80.08	19.56	24.42	56.10	60.65	84.65	95.35	113.60
	2012	78.97	18.84	23.86	56.50	59.95	81.65	94.40	111.90

**Table 8:** Summary statistics for lung cancer incidence rates in the Mid-South states, 1999–2012 (cases per 100,000).

State	Mean	Std Dev	CV(%)	Minimum	Lower Quartile	Median	Upper Quartile	Maximum
**Alabama**	76.66	30.52	39.81	33.10	46.30	75.55	106.95	118.20
**Arkansas**	82.82	31.31	37.80	33.40	56.70	79.30	112.45	134.50
**Kentucky**	108.36	31.00	28.61	65.80	80.45	102.05	139.30	173.30
**Louisiana**	83.83	31.04	37.02	46.50	55.65	74.10	109.80	150.20
**Mississippi**	83.91	32.02	38.16	45.40	53.60	80.45	111.05	138.90
**Tennessee**	83.00	25.82	31.11	48.00	60.20	78.55	105.50	125.70

**Table 9:** Summary statistics for lung cancer incidence rates for Blacks and Whites in the Mid-South by gender, 1999–2012 (cases per 100,000).

Race	Gender	Mean	StdDev	CV(%)	Minimum	Lower Quartile	Median	Upper Quartile	Maximum
**Black**	Female	53.61	15.15	28.25	33.10	44.30	50.05	56.10	93.40
	Male	121.30	16.77	13.83	91.30	108.50	119.45	130.70	173.30
**White**	Female	63.28	7.93	12.53	48.70	58.60	60.75	63.80	82.90
	Male	109.21	13.60	12.45	84.60	100.20	107.65	115.00	146.10

For both genders, the Mid-South had the highest prevalence of daily smokers among all U.S. regions, while New England had the lowest (**Table 10**).

**Table 10:** Summary statistics of mean percentage of daily smokers for males and females in 8 U.S. geographic regions, 1999–2012.

U.S. Region	Gender	Mean	Std Dev	Median	Minimum	Maximum	Confidence Interval
**Mid-Atlantic**	Male	20.28	3.41	34.84	14.03	27.40	(19.36,21.20)
	Female	17.88	2.65	18.70	11.51	21.57	(17.16,18.60)
**Mid- South**	Male	24.89	2.49	24.45	20.89	31.99	(24.27,25.51)
	Female	21.23	2.62	20.19	16.89	26.81	(20.60,21.89)
**Midwest**	Male	21.54	2.60	21.39	16.64	27.81	(21.12,21.96)
	Female	19.08	2.33	18.71	14.55	23.73	(18.68,19.48)
**New England**	Male	16.65	1.65	16.59	13.78	19.28	(16.03,17.28)
	Female	14.87	2.01	14.94	11.74	17.92	(14.18,15.56)
**Pacific Coast**	Male	17.56	1.89	17.71	13.89	21.11	(16.96,18.16)
	Female	14.97	2.21	15.01	10.84	18.98	(14.14,15.79)
**Rocky Mountain**	Male	19.44	3.01	18.76	14.84	25.04	(18.22,20.65)
	Female	17.57	3.10	16.16	12.79	22.54	(16.32,18.82)
**South**	Male	22.22	2.74	22.65	15.37	27.10	(21.70,22.74)
	Female	18.39	2.50	18.27	13.38	24.87	(17.90,18.88)
**Southwest**	Male	20.68	2.54	20.40	16.28	25.39	(19.89,21.47)
	Female	17.78	2.91	17.17	13.16	23.23	(16.87,18.69)

In the Mid-South, Kentucky had the highest percent of daily smokers for both genders, and Mississippi had the lowest percent of daily smokers for both genders (**Table 11**). Among all 38 U.S. states and District of Columbia, Kentucky had the highest percent of daily smokers for both races and genders, and California had the lowest percent.

**Table 11:** Summary statistics for mean percent of daily smokers in the Mid-South states, 1999–2012.

Mid-South State		Gender		Mean		Std Dev		Median		Minimum		Maximum		Confidence Interval	
**Alabama**		Male		23.25		1.30		23.57		20.89		24.83		(22.50,24.00)	
		Female		18.83		1.10		18.98		16.89		20.06		(18.20,19.47)	
**Arkansas**		Male		24.26		1.34		24.11		22.04		26.38		(23.41,25.12)	
		Female		21.06		1.12		21.02		19.15		22.81		(20.35,21.77)	
**Kentucky**		Male		28.29		2.60		28.23		24.43		31.99		(26.78,29.79)	
		Female		25.28		1.36		25.42		22.84		26.81		(24.50,26.07)	
**Louisiana**		Male		24.16		1.15		24.22		21.98		25.99		(23.50,24.83)	
		Female		19.45		0.56		19.65		18.57		20.29		(19.13,19.78)	
**Mississippi**		Male		23.03		1.40		23.08		20.79		25.04		(22.03,24.04)	
		Female		17.78		1.08		18.19		15.95		18.77		(17.00,18.55)	
**Tennessee**		Male		24.18		1.70		23.96		21.85		26.52		(22.97,25.40)	
		Female		21.63		1.45		21.63		19.79		23.28		(20.59,22.67)	

**Fig 3:**
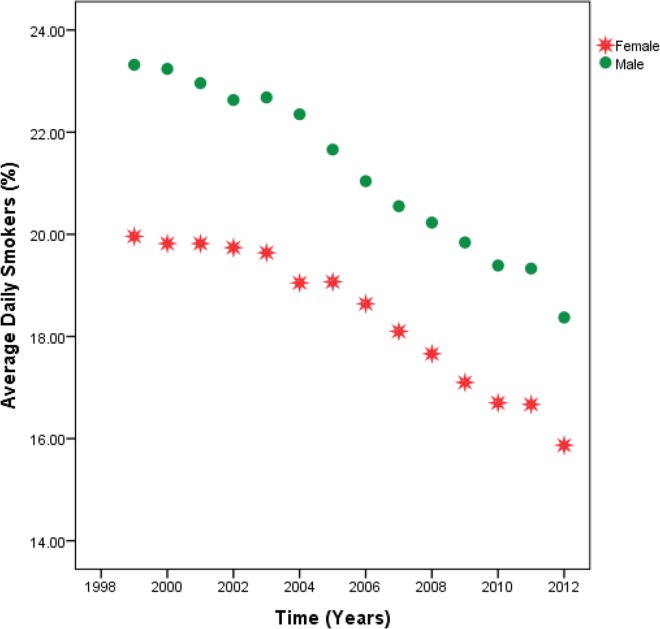
Estimates of mean percentage of daily smokers by gender across all U.S. geographic regions, 1999–2012.

**Fig 3**and **Table 12**show a downward trend in the prevalence of daily smokers in both genders. However, males have significantly higher rates of cigarette smoking than females at all time points.

**Table 12:** Summary statistics of the mean percent of daily males and females smokers for 38 states and District of Columbia, 1999–2012.

Gender	Year	Mean	Std Dev	Median	Minimum	Maximum	Confidence Interval
**Male**	1999	23.32	3.08	23.16	18.58	31.99	(22.25,24.40)
	2000	23.14	3.20	23.24	18.13	31.74	(22.02,24.26)
	2001	22.96	3.03	23.21	18.07	30.81	(21.92,24.00)
	2002	22.63	2.91	23.16	17.72	29.36	(21.62,23.65)
	2003	22.68	3.20	22.94	17.19	30.77	(21.63,23.73)
	2004	22.35	3.11	22.46	16.85	30.17	(21.31,23.39)
	2005	21.66	3.09	21.69	16.35	29.41	(20.65,22.68)
	2006	21.04	2.92	20.95	15.83	27.09	(20.08,22.00)
	2007	20.55	2.90	20.59	15.33	26.93	(19.59,21.50)
	2008	20.23	2.90	20.22	15.16	26.75	(19.27,21.18)
	2009	19.84	2.94	19.83	14.75	25.79	(18.86,20.82)
	2010	19.39	2.85	19.16	14.49	25.19	(18.45,20.32)
	2011	19.33	3.02	18.82	14.49	25.57	(18.32,20.33)
	2012	18.37	2.96	18.15	13.78	24.43	(17.40,19.34)
**Female**	1999	19.96	2.65	19.84	15.02	26.67	(18.99,20.93)
	2000	19.82	2.67	19.68	14.81	26.81	(18.86,20.78)
	2001	19.82	2.51	19.61	15.57	26.58	(18.90,20.74)
	2002	19.74	2.67	19.47	14.43	25.96	(18.77,20.70)
	2003	19.64	2.37	19.29	15.72	26.71	(18.83,20.45)
	2004	19.05	2.61	18.74	13.69	26.36	(18.15,19.94)
	2005	19.07	2.81	18.71	13.36	26.27	(18.11,20.04)
	2006	18.64	2.77	18.54	13.00	24.69	(17.69,19.59)
	2007	18.10	2.76	17.90	12.64	24.74	(17.20,19.00)
	2008	17.66	2.89	17.51	12.42	24.88	(16.70,18.62)
	2009	17.10	2.75	17.01	11.98	24.27	(16.17,18.02)
	2010	16.70	2.91	16.38	11.59	23.42	(15.74,17.65)
	2011	16.67	3.03	16.46	11.32	23.77	(15.68,17.67)
	2012	15.87	2.80	15.90	10.84	22.84	(14.92,16.82)

Among the 8 U.S. geographic regions, the Mid-South had the highest lung cancer incidence rate as well as the highest percentage of daily smokers (**Table 13**). The estimated correlation coefficient between lung cancer incidence rates and the percentage of daily smokers for the 8 U.S. geographic regions was 0.74, indicating a strong influence of cigarette smoking on regional incidence rates of lung cancer.

**Table 13:** Summary statistics of average lung cancer incidence rates and average daily smokers in percentage in 8 U.S. geographic regions, 1999–2012.

U.S. Region	Incidence Rate (cases per 100,000)	Daily Smokers (%)
**Rocky Mountain**	63.72	18.51
**New England**	69.25	15.76
**Mid-Atlantic**	69.28	19.08
**Pacific Coast**	72.59	16.27
**Southwest**	73.56	19.23
**South**	76.07	20.31
**Midwest**	81.63	20.31
**Mid-South**	86.85	23.06

## Discussion

This longitudinal study assessed differences in lung cancer incidence rates between Black and White males and females in 38 U.S. states, the District of Columbia, and eight U.S. geographic regions from 1999 to 2012. Using a longitudinal linear mixed-effects model, we demonstrated that age-adjusted incidence rates in lung cancer have decreased across the U.S., but racial and gender disparities persist. Although the racial gap has decreased over time, Blacks continue to have higher age-adjusted incidence rates for lung cancer than Whites, with these racial disparities being significantly worse among men than women. Black males bear the highest burden of lung cancer incidence of all subgroups, followed by White males, White females, and Black females. Importantly, our model predicts that the racial gap in lung cancer incidence among males will not disappear in the near future. In contrast, the gender gap will gradually disappear–by mid-2018 for Whites and by 2026 for Blacks–provided current lung cancer incidence rates remain within reasonable range.

The study revealed a strong association between lung cancer incidence rates and prevalence of cigarette smoking. Among all U.S. geographic regions, regardless of race and gender the Mid-South has both the highest overall lung cancer incidence rate (86.85) and percentage of daily smokers (23.06). Although there is a clear downward trend in cigarette smoking in both genders, males continue to have significantly higher rates of cigarette smoking than females at all time points, which is reflected in their higher lung cancer incidence rates at all time points. These findings are consistent with previous research, which attributes racial and gender disparities in lung cancer incidence rates to differences in tobacco use [27, 28].

In addition to tobacco use, lung cancer incidence is associated with environmental and occupational exposures, family history, stage at diagnosis, and a number of psycho-social factors [27, 29–32]. While the National Lung Screening Trial showed a 20% reduction in risk of death from lung cancer in high-risk patients screened with low-dose CT as opposed to chest radiography [33], no screening test has been shown to decrease incidence or mortality rates of lung cancer in the general population [34]. In the absence of viable screening options, prevention efforts need to focus on major population risk factors, such as tobacco use.

It has been established that tobacco use is associated with socioeconomic status [35–37]. For example, Hu et al. report higher use of tobacco products among persons with a GED certificate or less than high-school education and those with annual household income <$20,000 [4]; they also observe higher prevalence of cigarette smoking in the Midwest and the South, which is corroborated by the findings of our study. Considering such evidence, approaches that may help reduce lung cancer incidence rates include [26, 28]: 1) Counseling through tobacco quit lines and free nicotine replacement therapy; 2) Media campaigns to discourage initiation of smoking, encourage smoking cessation, and protect nonsmokers from second-hand smoke; 3) Tobacco and vapor-free policies in institutions and recreation facilities; 4) Reinforcing comprehensive smoking bans on airlines and in buildings; 5) Improved health coverage of smoking cessation treatments for all smokers, especially for pregnant women, federal employees, retirees, and their spouses and dependents; 6) Free radon tests in homes; 7) Continued surveillance of lung cancer incidence and smoking prevalence within racial and ethnic groups in the U.S.

The model presented in this paper accurately describes the dynamics of lung cancer incidence rates by race, gender, and smoking prevalence across the United States from 1999 to 2012. Previous research shows that lung cancer incidence varies also by histology: while squamous, large, and small cell carcinoma rates continue to decrease for all gender-race combinations, adenocarcinoma rates remain relatively constant in males and are increasing in females [38]. Future investigation should therefore include additional covariates of lung cancer incidence, such as histological type and tumor size. To the best of our knowledge, this work is the first attempt to analyze disparities in lung cancer in the United States using a longitudinal linear mixed-effects model. The developed model could help health professionals and policy makers make predictions about age-adjusted lung cancer incidence rates for approximately five to ten years after 2012.
